# Association between epidermal growth factor (EGF) and EGF receptor gene polymorphisms and end-stage renal disease and acute renal allograft rejection in a Korean population 

**DOI:** 10.1080/0886022X.2019.1710535

**Published:** 2020-01-06

**Authors:** Byeong Woo Kim, Su Kang Kim, Kyung Wook Heo, Ki Beom Bae, Kyung Hwan Jeong, Sang Ho Lee, Tae Hee Kim, Yeong Hoon Kim, Sun Woo Kang

**Affiliations:** a Department of Internal Medicine, Haeundae Bumin Hospital, Busan, Korea; b Kohwang Medical Research Institute, Kyung Hee University School of Medicine, Seoul, Korea; c Department of Otolaryngology, Inje University, Busan, Korea; d Department of General Surgery, Inje University, Busan, Korea; e Division of Nephrology, Department of Internal Medicine, Kyung Hee University, Seoul, Korea; f Division of Nephrology, Department of Internal Medicine, Inje University, Busan, Korea

**Keywords:** Epidermal growth factor, epidermal growth factor receptor, single nucleotide polymorphism, kidney transplantation, graft rejection, end stage renal disease

## Abstract

**Purpose:**

Epidermal growth factor (EGF) has been found to be associated with the development and repair mechanisms of several renal diseases. In this study, we hypothesized that single nucleotide polymorphisms (SNPs) in EGF or its receptor genes might have an association with end-stage renal disease (ESRD) or acute renal allograft rejection (AR) in a Korean population.

**Methods:**

Three-hundred and forty seven recipients of the first renal transplants for ESRD, including 63 AR patients along with 289 healthy adults were included in the study. Five EGF gene SNPs (rs11568835, rs11568943, rs2237051, rs11569017, and rs3756261) and four EGFR gene SNPs (rs1140475, rs2293347, rs1050171, and rs6965469) were analyzed. The genotypes of these SNPs were analyzed using the AxiomTM genome-wide human assay. Statistical analysis was performed using SNPStats and Haploview version 4.2 software. Multiple logistic regression models (codominant, dominant, recessive, and Log-additive) were used to estimate the odds ratio (OR), 95% confidence interval (CI), and P value.

**Results:**

One SNP (rs11569017) in the EGF gene showed significant association with ESRD but not with AR. Another SNP (rs11568835) in the EGF gene showed significant association with susceptibility to AR but not with ESRD. One SNP (rs1050171) in the EGFR gene showed significant association with susceptibility to AR but not with ESRD.

**Conclusion:**

Our findings suggest that SNPs in the EGF and EGFR gene may be associated with the risk of ESRD and AR development in the Korean population.

## Introduction

Renal transplantation is the treatment of choice for patients with end stage renal disease (ESRD). Renal transplantation improves the quality of life as well as the long-term survival of patients [1,2]. However, the graft loss leads to a sudden decrease in the anticipated improvement in life span [3]. Acute renal allograft rejection (AR) is the single most important risk factor for developing chronic renal allograft rejection [4,5]. Numerous genetic polymorphisms have been determined to be associated with AR, including those in human leukocyte antigens (HLA) [6]. Despite the advances made in immunosuppression and technical care of renal transplant recipients, leading to an improvement in the first-year allograft survival, chronic renal allograft rejection continues to be a major impediment over the decades [7–9].

Epidermal growth factor (EGF), a canonical ligand with the highest affinity to EGF receptor (EGFR), has been reported to be involved in the modulation of tissue response to injury in the kidney after tubulointerstitial damage [10,11]. Following acute kidney injury (AKI), EGF produced by the proximal tubule cells stimulates vascular epidermal growth factor secretion and enhances proliferation of proximal tubule cells, which is important for the recovery from AKI [12].

In animal models, a significant decrease in the expression of EGF mRNA has been observed in acute and chronic renal allograft rejection. The distribution of EGF mRNA was found to be localized to the distal convoluted tubule and the thick ascending limb of Henle’s loop in the controls and to the well-preserved areas of the tissue in chronic rejection model [13].

EGF gene located on chromosome 4q25-q27, contains 24 exons and 23 introns. This gene also encodes a member of the EGF superfamily. Following reports about polymorphism of 5’-untranslated region of the EGF gene (rs4444903; A61G) being associated with susceptibility or severity to several human malignancies, studies on EGF polymorphisms in the field of oncology have acquired increased attention in recent times [14,15].

EGFR, also known as HER1 and ERBB1, is a transmembrane glycoprotein of 1186 amino acids. EGFR expression is specifically localized in the glomerulus and the tubulointerstitial compartment of the kidney. EGFR plays a key role in renal homeostasis of electrolytes [16]. However, in pathological condition, both beneficial and deleterious roles of EGFR have been observed. In progressive kidney disease, fibrotic renal damage seems to be ameliorated by blocking EGFR [17,18]. However, in the AKI model, renal function recovery and tubular regeneration were both significantly delayed when treated with EGFR tyrosine kinase inhibitor or in the setting of EGFR mutation that induces a reduction in receptor tyrosine kinase activity [19,20].

Thirty years after EGF has been known to be involved in the regeneration of renal tubular epithelium, there have been few studies on whether the genetic variation of EGF or its receptor affects the development of ESRD or AR. Therefore, we investigated whether single nucleotide polymorphisms (SNPs) of EGF and EGFR gene are associated with ESRD or AR.

## Materials and methods

### Study subjects

A total of 347 first renal allograft recipients who underwent transplantation at two hospitals in Korea (Busan Paik Hospital, Busan and Kyung Hee University Medical Center, Seoul) from 1982 to 2009 were included. Demographic data of recipients and donors (age, gender, cause of ESRD, number of HLA-mismatch, the last serum creatinine of donor, presence of rejection episodes, and immunosuppressive therapy) were extracted from the hospital record. AR was determined by allograft biopsy or by the clinical increase in creatinine level by 30% from baseline, which was not attributable to other causes, with subsequent return to baseline after anti-rejection therapy. A total of 289 healthy control participants with no past medical history of renal disease were enrolled during regular checkups at the study hospitals from 2002 to 2005. The recruited participants were evenly distributed by age and sex (Table 1). The Institutional Review Board approved this study, and written informed consent was obtained at the time of enrollment.

**Table 1.** Age and sex distribution of the study subjects.

Age	10–19	20–29	30–39	40–49	50–59	60–69	∼70	Total
Control								
Male(*n*)			35	38	36	37	11	157
Female(*n*)			28	25	35	34	10	132
KT-NonAR								
Male(*n*)*	7	30	36	46	39	4	0	162
Female(*n*)*	2	22	24	44	28	2	0	122
KT-AR								
Male(*n*)*	1	9	13	15	5	3	0	46
Female(*n*)*	0	3	6	7	1	0	0	17

KT: kidney transplantation; AR: acute rejection; *n*: number.

^*^ Age when underwent kidney transplantation.

Peripheral blood samples were collected in EDTA tubes, and genomic DNA was extracted from the peripheral blood lymphocytes using a commercially available DNA extraction kit (Qiagen, Tokyo, Japan).

### SNP selection and genotyping

We first searched for EGF and EGFR genes containing SNPs in the National Center for Biotechnology Information (NCBI) database (http://www.nc. . . . . . .bi. nlm.nih.gov/gene/). The selection criteria for exonic, promoter, and intronic SNPs in each gene were as follows: (1) a minor allele frequency >10%; (2) heterozygosity >10%; (3) known genotype frequencies in Asian populations; and (4) been examined in a previous study. Five EGF gene SNPs (rs11568835, rs11568943, rs2237051, rs11569017, and rs3756261) and four EGFR gene SNPs (rs1140475, rs2293347, rs1050171, and rs6965469) were selected to investigate their association with ESRD and AR. Genotyping of all SNPs was carried out using an Axiom™ Genome-Wide Human Assay (Affymetrix, Inc., Santa Clara, CA, USA). All experimental procedures involved in this assay were performed at the Theragen Etex Corporation (Suwon, Korea) as per the manufacturer’s instruction.

### Statistical analysis

Compliance with the Hardy–Weinberg equilibrium was evaluated using the SNPstats software (http://bioinfo.iconcologia.net/index.php. . . . . . . ) for all the SNPs. All continuous variables were expressed as the mean ± standard deviation of the allelic frequencies and were assessed using the Chi-square test. For association tests, we calculated the odds ratios (ORs), 95% confidence intervals (CIs), and P values with SNPstats, HapAnalyzer version 1.0, and SNP analyzer (ISTECH, Inc., Goyang, Korea). The differences in genotype distribution between the ESRD group and the control group as well as the AR group and the non-AR group were analyzed using a multiple logistic regression test to adjust for age and sex. We used multiple inheritance models, including codominant 1 (major allele homozygotes vs. heterozygotes), codominant 2 (major allele homozygotes vs. minor allele homozygotes), dominant (major allele homozygotes vs. minor allele homozygotes + heterozygotes), recessive (major allele homozygotes + heterozygotes vs. minor allele homozygotes), and log-additive (major allele homozygotes vs. heterozygotes vs. minor allele homozygotes). The presence of a linkage disequilibrium block of polymorphisms was assessed using Haploview version 4.1 (Broad Institute of MIT and Harvard, Cambridge, MA, USA; http://www.broadinstitute.org/haploview/haploview. . . . . . . ). We also used the online program AliBaba2.1 (Labmom.com; http://www.gene-regulation.com/pub/. . . . . . . programs/alibaba2). Clinical characteristics were compared using the Chi-square test and Student’s unpaired t-test. *p* < .05 was considered statistically significant.

## Results

A total of 347 patients who had undergone first kidney transplant with or without episodes of acute renal allograft rejection and 289 healthy controls were studied. Table 1 shows the age and sex distribution of the subjects. Individuals in the control group were at least 30 years old and had been recruited during their regular health screenings. The mean age was higher in the control group than in the test group (51.9 ± 12.6 in control group, 40.2 ± 11.4 in all kidney transplantation patients). Table 2 shows the clinical characteristics of the non-AR and the AR groups. The proportion of males and the mean age of donor was higher in the AR group (39.4 ± 14 vs. 44.8 ± 13.7). The proportion of deceased donors and Basiliximab induction were higher in the non-AR group (28.87% vs. 19.05% for deceased donors; 88.4% vs. 11.6% for Basiliximab induction). Donor age, renal function of donor, total number of HLA mismatch, proportion of calcineurin inhibitor prescription, cyclosporin or tacrolimus-based regimen, and causes of ESRD were similar in the non-AR and the AR groups. In this study, glomerulonephritis was the most common cause of ESRD (40.1% and 42.9%), followed by hypertension (27.5% and 27%) and diabetes mellitus (10.9% and 6.3%) in both groups.

**Table 2.** Clinical characteristics of patients with and without acute rejection episodes.

	Non-AR	AR	*p* Value
Subject (*n*)	284	63	
Age*	40.5 ± 11.5	39 ± 10.5	.345
Male (%)	57.04%	73.00%	.019
Donor age	39.4 ± 14	44.8 ± 13.7	.012
Deceased donors	28.87%	19.05%	.02
HLA-mismatch (total number)	3.2 ± 1.6	3.4 ± 1.2	.416
Calcineurin inhibitors	98.94%	98.41%	.738
Cyclosporin-based regimen	78.9% (172)	21.1%(46)	.096
Tacrolimus-based regimen	86.3% (101)	25.4% (16)	.099
Basiliximab induction	88.4% (167)	11.6% (22)	.001
Donor serum Cr (mg/dL)	1.1 ± 0.6	0.9 ± 0.3	.094
Cause of ESRD			.929
GN	114 (40.1)	27 (42.9)	
HTN	78 (27.5)	17 (27)	
DM	31 (10.9)	4 (6.3)	
PCKD	5 (1.8)	1 (1.6)	
Unknown	43 (15.1)	11 (17.5)	
Others	13 (4.6)	3 (4.8)	

AR: acute rejection; ESRD: end stage renal disease; DM: diabetes mellitus; HLA: human leukocyte antigen; HTN: hypertension; GN: glomerulonephritis; PCKD: polycystic kidney disease; n: number; Cr: creatinine.

^*^ Age when underwent kidney transplantation.

The genotype distributions of the five SNPs of the EGF gene (rs11568835, rs11568943, rs2237051, rs11569017, and rs3756261) in patients who had undergone renal transplantation and the control subjects are shown in Table 3. The genotype distributions of the four SNPs of the EGFR gene (rs1140475, rs2293347, rs1050171, and rs6965469) in patients who had undergone renal transplantation and the control subjects are shown in Table 4. Tables 3 and 4 also show the relation between each genotype and the risk of susceptibility to ESRD by logistic regression analysis after being adjusted for age and sex. Among the nine SNPs, TT genotype of one polymorphism in the EGF gene (rs11569017) was found to be associated with susceptibility to ESRD.

**Table 3.** Genotype frequencies of polymorphisms of EGF gene in ESRD patients undergone kidney transplantation and controls.

SNP	Genotype	Control	KT	Models	OR (95% CI)	*p* Value
		*n* (%)	*n* (%)			
rs11568835	G/G	195 (67.5%)	222 (64%)	Codominant1	1.13 (0.80–1.59)	.50
	A/G	85 (29.4%)	109 (31.4%)	Codominant2	1.57 (0.68–3.61)	.30
	A/A	9 (3.1%)	16 (4.6%)	Dominant	1.15 (0.83–1.60)	.41
				Recessive	1.44 (0.62–3.31)	.39
				Log-additive	1.15 (0.87–1.53)	.32
rs11568943	G/G	193 (66.8%)	220 (63.4%)	Codominant1	1.13 (0.81–1.59)	.48
	A/G	86 (29.8%)	111 (32%)	Codominant2	1.40 (0.62–3.17)	.41
	A/A	10 (3.5%)	16 (4.6%)	Dominant	1.17 (0.84–1.63)	.35
				Recessive	1.34 (0.60–3.00)	.48
				Log-additive	1.16 (0.88–1.53)	.3
rs2237051	A/A	131 (45.3%)	169 (48.7%)	Codominant1	0.85 (0.61–1.17)	.32
	A/G	132 (45.7%)	144 (41.5%)	Codominant2	0.01 (0.58–1.77)	.96
	G/G	26 (9%)	34 (9.8%)	Dominant	0.86 (0.63–1.18)	.35
				Recessive	1.10 (0.64–1.88)	.74
				Log-additive	0.93 (0.73–1.19)	.58
rs11569017	A/A	184 (63.9%)	216 (62.2%)	Codominant1	0.96 (0.69–1.33)	.80
	T/A	104 (36.1%)	117 (33.7%)	Codominant2	NA (0.00–NA)	<.0001
	T/T	0 (0%)	14 (4%)	Dominant	1.09 (0.78–1.50)	.62
				Recessive	NA (0.00–NA)	<.0001
				Log-additive	1.24 (0.92–1.67)	.16
rs3756261	A/A	188 (65%)	217 (62.7%)	Codominant1	1.10 (0.78–1.53)	.62
	A/G	89 (30.8%)	112 (32.4%)	Codominant2	1.23 (0.57–2.64)	.60
	G/G	12 (4.2%)	17 (4.9%)	Dominant	1.12 (0.81–1.55)	.50
				Recessive	1.19 (0.56–2.53)	.66
				Log-additive	1.11 (0.84–1.45)	.47

EGF: epidermal growth factor; ESRD: end stage renal disease; KT: kidney transplantation.

**Table 4.** Genotype frequencies of polymorphisms of EGFR gene in ESRD patients undergone kidney transplantation and controls.

SNP	Genotype	Control	KT	Models	OR (95% CI)	*p* Value
		*n* (%)	*n* (%)			
rs1140475	C/C	255 (88.2%)	315 (90.8%)	Codominant1	0.74 (0.44–1.23)	.25
	T/C	34 (11.8%)	31 (8.9%)	Codominant2	NA (0.00–NA)	1.00
	T/T	0 (0%)	1 (0.3%)	Dominant	0.76 (0.45–1.26)	.28
				Recessive	NA (0.00–NA)	.28
				Log-additive	0.79 (0.48–1.30)	.35
rs2293347	G/G	128 (44.3%)	153 (44.1%)	Codominant1	0.97 (0.70–1.35)	.86
	A/G	132 (45.7%)	153 (44.1%)	Codominant2	1.18 (0.70–2.01)	.54
	A/A	29 (10%)	41 (11.8%)	Dominant	1.01 (0.74–1.38)	.96
				Recessive	1.20 (0.73–2.00)	.47
				Log-additive	1.05 (0.83–1.32)	.71
rs1050171	G/G	221 (76.5%)	264 (76.1%)	Codominant1	1.04 (0.71–1.51)	.85
	A/G	63 (21.8%)	78 (22.5%)	Codominant2	0.84 (0.24–2.92)	.78
	A/A	5 (1.7%)	5 (1.4%)	Dominant	1.01 (0.70–1.46)	.96
				Recessive	0.87 (0.25–3.04)	.82
				Log-additive	1.00 (0.71–1.39)	.99
rs6965469	C/C	179 (62.1%)	233 (67.3%)	Codominant1	0.79 (0.56–1.10)	.16
	T/C	101 (35.1%)	103 (29.8%)	Codominant2	0.96 (0.37–2.48)	.93
	T/T	8 (2.8%)	10 (2.9%)	Dominant	0.81 (0.58–1.12)	.21
				Recessive	1.05 (0.41–2.70)	.92
				Log-additive	0.85 (0.64–1.14)	.28

EGFR: epidermal growth factor receptor; ESRD: end stage renal disease; KT: kidney transplantation.

The genotype distribution of the nine SNPs of the EGF and EGFR genes in patients with and without an episode of acute renal allograft rejection are shown in Tables 5 and 6. Tables 5 and 6 also show the relation between each genotype and the risk of susceptibility to AR by logistic regression analysis. One polymorphism from EGF gene (rs11568835) and another polymorphism from EGFR gene (rs1050171) were found to be associated with the susceptibility to AR after being adjusted for age and sex. These associations were also observed after additional adjusting for donor age, donor type, number of HLA mismatch, cyclosporin or tacrolimus-based immunosuppression, and Basiliximab induction (Tables 7 and 8).

**Table 5.** Genotype frequencies of polymorphisms of EGF gene in kidney transplant patients with and without acute rejection episodes.

SNP	Genotype	Non-AR	AR	Models	OR (95% CI)	*p* Value
		*n* (%)	*n* (%)			
rs11568835	G/G	175 (61.6%)	47 (74.6%)	Codominant1	0.64 (0.34–1.20)	.16
	A/G	93 (32.8%)	16 (25.4%)	Codominant2	0.00 (0.00–NA)	.047
	A/A	16 (5.6%)	0 (0%)	Dominant	0.52 (0.28–0.97)	.034
				Recessive	0.00 (0.00–NA)	.0076
				Log-additive	0.50 (0.28–0.88)	.0099
rs11568943	G/G	181 (63.7%)	39 (61.9%)	Codominant1	1.02 (0.56–1.85)	.95
	A/G	91 (32%)	20 (31.8%)	Codominant2	1.55 (0.47–5.05)	.47
	A/A	12 (4.2%)	4 (6.3%)	Dominant	1.10 (0.62–1.94)	.75
				Recessive	1.53 (0.47–4.95)	.5
				Log-additive	1.13 (0.71–1.80)	.6
rs2237051	A/A	140 (49.3%)	29 (46%)	Codominant1	1.11 (0.63–1.99)	.72
	A/G	117 (41.2%)	27 (42.9%)	Codominant2	1.25 (0.50–3.15)	.63
	G/G	27 (9.5%)	7 (11.1%)	Dominant	1.08 (0.62–1.87)	.8
				Recessive	1.20 (0.49–2.93)	.69
				Log-additive	1.08 (0.72–1.64)	.7
rs11569017	A/A	178 (62.7%)	38 (60.3%)	Codominant1	1.03 (0.57–1.85)	.94
	T/A	96 (33.8%)	21 (33.3%)	Codominant2	1.87 (0.56–6.30)	.31
	T/T	10 (3.5%)	4 (6.3%)	Dominant	1.15 (0.65–2.02)	.63
				Recessive	2.02 (0.60–6.78)	.28
				Log-additive	1.21 (0.76–1.95)	.43
rs3756261	A/A	180 (63.6%)	37 (58.7%)	Codominant1	1.19 (0.66–2.14)	.56
	A/G	90 (31.8%)	22 (34.9%)	Codominant2	1.50 (0.46–4.85)	.50
	G/G	13 (4.6%)	4 (6.3%)	Dominant	1.26 (0.72–2.22)	.42
				Recessive	1.43 (0.45–4.60)	.56
				Log-additive	1.23 (0.78–1.94)	.37

EGF: epidermal growth factor; AR: acute rejection.

**Table 6.** Genotype frequencies of polymorphisms of EGFR gene in kidney transplant patients with and without acute rejection episodes.

SNP	Genotype	Non-AR	AR	Models	OR (95% CI)	*p* Value
		*n* (%)	*n* (%)			
rs1140475	C/C	257 (90.5%)	58 (92.1%)	Codominant1	0.85 (0.31–2.31)	.75
	T/C	26 (9.2%)	5 (7.9%)	Codominant2	0.00 (0.00–NA)	1.00
	T/T	1 (0.4%)	0 (0%)	Dominant	0.77 (0.28–2.10)	.60
				Recessive	0.00 (0.00–NA)	.47
				Log-additive	0.75 (0.28–1.97)	.54
rs2293347	G/G	127 (44.7%)	26 (41.3%)	Codominant1	1.01 (0.55–1.85)	.43
	A/G	127 (44.7%)	26 (41.3%)	Codominant2	1.00 (0.55–1.82)	1.00
	A/A	30 (10.6%)	11 (17.5%)	Dominant	1.15 (0.66–2.01)	.62
				Recessive	1.68 (0.78–3.60)	.20
				Log-additive	1.23 (0.82–1.83)	.32
rs1050171	G/G	223 (78.5%)	41 (65.1%)	Codominant1	2.14 (1.18–3.87)	.012
	A/G	56 (19.7%)	22 (34.9%)	Codominant2	0.00 (0.00–NA)	.60
	A/A	5 (1.8%)	0 (0%)	Dominant	1.86 (1.02–3.39)	.046
				Recessive	0.00 (0.00–NA)	.15
				Log-additive	1.55 (0.90–2.68)	.12
rs6965469	C/C	189 (66.8%)	44 (69.8%)	Codominant1	0.73 (0.39–1.39)	.34
	T/C	88 (31.1%)	15 (23.8%)	Codominant2	2.86 (0.78–10.58)	.12
	T/T	6 (2.1%)	4 (6.3%)	Dominant	0.89 (0.49–1.62)	.71
				Recessive	3.80 (0.99–14.60)	.065
				Log-additive	1.08 (0.65–1.81)	.77

EGFR: epidermal growth factor receptor; AR: acute rejection.

**Table 7.** Genotype frequencies of polymorphisms of EGF gene in kidney transplant patients with and without acute rejection episodes adjusted by patient age, sex, donor age, deceased donor, cyclosporin, tacrolimus, basiliximab and HLA mismatch.

SNP	Genotype	Non-AR	AR	Models	OR (95% CI)	*p*
		*n* (%)	*n* (%)			
rs11568835	G/G	124 (62.9%)	38 (73.1%)	Dominant	0.49 (0.24–1.02)	.05
	A/G	59 (29.9%)	14 (26.9%)	Recessive	0.00 (0.00–NA)	.0014
	A/A	14 (7.1%)	0 (0%)	Log-additive	0.45 (0.24–0.85)	.0083
rs11568943	G/G	129 (65.5%)	34 (65.4%)	Dominant	0.94 (0.47–1.89)	.87
	A/G	60 (30.5%)	16 (30.8%)	Recessive	1.28 (0.24–6.86)	.77
	A/A	8 (4.1%)	2 (3.8%)	Log-additive	0.99 (0.55–1.78)	.96
rs2237051	A/A	99 (50.2%)	22 (42.3%)	Dominant	1.25 (0.65–2.41)	.5
	A/G	84 (42.6%)	23 (44.2%)	Recessive	2.29 (0.80–6.50)	.13
	G/G	14 (7.1%)	7 (13.5%)	Log-additive	1.36 (0.83–2.25)	.23
rs11569017	A/A	128 (65%)	33 (63.5%)	Dominant	1.06 (0.53–2.10)	.87
	T/A	62 (31.5%)	16 (30.8%)	Recessive	2.16 (0.48–9.70)	.33
	T/T	7 (3.5%)	3 (5.8%)	Log-additive	1.15 (0.65–2.05)	.63
rs3756261	A/A	127 (64.5%)	33 (63.5%)	Dominant	0.96 (0.48–1.91)	.91
	A/G	61 (31%)	17 (32.7%)	Recessive	1.07 (0.21–5.49)	.94
	G/G	9 (4.6%)	2 (3.8%)	Log-additive	0.98 (0.55–1.75)	.94

EGF: epidermal growth factor; AR: acute rejection; HLA: human leukocyte antigen.

**Table 8.** Genotype frequencies of polymorphisms of EGFR gene in kidney transplant patients with and without acute rejection episodes adjusted by patient age, sex, donor age, deceased donor, cyclosporin, tacrolimus, basiliximab and HLA mismatch.

SNP	Genotype	Non-AR	AR	Models	OR (95% CI)	*p*
		*n* (%)	*n* (%)			
rs1140475	C/C	174 (88.3%)	48 (92.3%)	Dominant	0.40 (0.12–1.33)	.11
	T/C	22 (11.2%)	4 (7.7%)	Recessive	0.00 (0.00–NA)	.39
	T/T	1 (0.5%)	0 (0%)	Log-additive	0.41 (0.13–1.29)	.097
rs2293347	G/G	84 (42.6%)	22 (42.3%)	Dominant	1.08 (0.56–2.08)	.83
	A/G	96 (48.7%)	23 (44.2%)	Recessive	2.23 (0.80–6.21)	.14
	A/A	17 (8.6%)	7 (13.5%)	Log-additive	1.25 (0.75–2.08)	.39
rs1050171	G/G	154 (78.2%)	32 (61.5%)	Dominant	2.74 (1.30–5.74)	.0082
	A/G	41 (20.8%)	20 (38.5%)	Recessive	0.00 (0.00–NA)	.42
	A/A	2 (1%)	0 (0%)	Log-additive	2.40 (1.19–4.83)	.016
rs6965469	C/C	132 (67%)	35 (67.3%)	Dominant	1.03 (0.51–2.07)	.93
	T/C	61 (31%)	13 (25%)	Recessive	2.93 (0.63–13.71)	.18
	T/T	4 (2%)	4 (7.7%)	Log-additive	1.18 (0.67–2.10)	.57

EGFR: epidermal growth factor receptor; AR: acute rejection; HLA: human leukocyte antigen.

**Figure 1.**
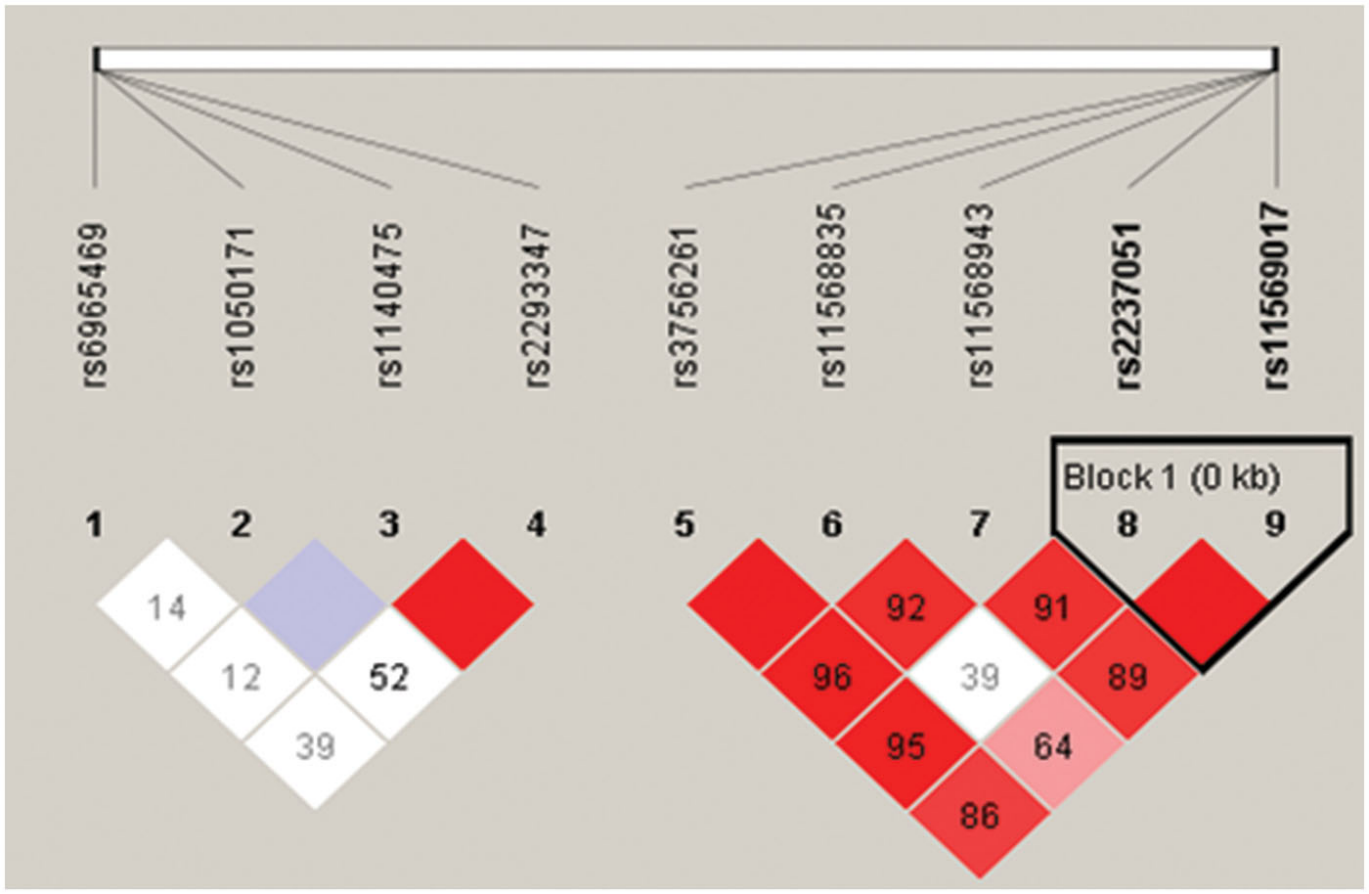
LD block composed of SNPs of EGF and EGFR in kidney transplant patients with and without acute rejection episodes. Linkage disequilibrium block composed of single nucleotide polymorphisms of epidermal growth factor and epidermal growth factor receptor genes in kidney transplant patients with and without acute rejection episodes.

Nine SNPs were analyzed by linkage disequilibrium (LD) and haplotype. One LD block was constructed by the Gabriel method during pair-wise comparison among these SNPs (Figure 1). The LD block comprised of rs2237051 and rs11569017 of the EGF gene. However, we did not detect significant association between the haplotype and AR (Table 9).

**Table 9.** Haplotype analysis for association between the EGF gene cluster polymorphisms and acute renal allograft rejection.

Haplotype	Haplotype frequency	Non AR		AR		Case, Control Ratio Counts	Chi square	*p* value
		+	−	+	−			
AA	0.486	280	286	56	70	0.444, 0.495	1.042	.3074
GA	0.305	170	396	41	85	0.325, 0.300	0.305	.5808
AT	0.210	116	450	29	97	0.230, 0.205	0.395	.5294

EGF: epidermal growth factor; AR: acute rejection.

## Discussion

According to a study by Ju *et al.*, EGF has a high kidney-specific expression compared to that in 84 other human organs or tissues including lung, heart, and liver and blood; it is especially concentrated in the renal cortex and medulla than in the glomeruli, papillary tips, or pelvis. Decreased urinary EGF-to-creatinine ratio, which reflects decreased intra-renal EGF transcript expression, shows a higher correlation with the estimated glomerular filtration rate (eGFR) slope than with the urinary albumin-to-creatinine ratio with eGFR slope. They suggested urinary EGF as a prognostic biomarker for progression of chronic kidney disease [21].

Tubulointerstitial injury is an invariant finding in the chronically diseased kidney, irrespective of the type of disease. While glomerular lesions can be reversible, tubular injury is closely associated with the progression of nephropathies and decline of renal function [22]. Tubular injury, frequently observed in renal allografts in the early post transplantation period, is an important component in the diagnosis of AR, especially acute T-cell mediated rejection [23]. Tubular proteins, such as EGF may be better predictive markers than proteinuria or albuminuria for the prognosis of not only glomerulonephritis but also renal transplantation in patients.

In this study, we first compared EGF polymorphisms between patients who underwent renal transplantation and healthy controls. Although patients who had undergone kidney transplantation were not thought to be representative of ESRD patients, they are ESRD patients, but tend to have been diagnosed with ESRD at a younger age. This suggests that there are underlying genetic causes associated with ESRD.

We found that the TT genotype of rs11569017 of the EGF gene was found in only the ESRD patients but not in the control group. However, this SNP seems to not be associated with AR. The TT genotype of rs11569017 was reported to be associated with the risk of hepatitis B virus-related hepatocellular carcinoma in Chinese population [24]. Tian et al. reported that the frequency of TT genotype of rs11569017 was significantly higher in major depressive disorder patients than that in the control group in the Chinese population. They also showed decreased secretion of EGF in the HEK293 cells that express the rs11569017 mutant [25]. Kim *et al.* studied the association between EGF, EGFR polymorphisms, and benign prostatic hyperplasia in the Korean population. They found that two SNPs of the EGF gene (rs11568943 and rs11569017) were significantly associated with prostate volume, while three SNPs of the EGF gene (rs37566261, rs11568943, and rs11569017) and rs2293347 of the EGFR gene were associated with serum PSA level [26].

The rs11569017 SNP is an exonic non-synonymous missense variant SNP (D784V). The locus of this SNP is included in the precursor EGF (prepro EGF) composed of 1207-amino acids, but disappears during the proteolytic cleaving process to generate the 53-amino acid EGF peptide. Prepro EGF is synthesized as a membrane-bound protein and has a region homologous to the low-density lipoprotein receptor [27,28]. Hence, it has been proposed that the prepro EGF may function as a membrane receptor for an unidentified ligand [29]. This non-synonymous exonic SNP may increase the susceptibility to ESRD by a functional change of prepro EGF.

One SNP (rs11568835) located in the promoter region of EGF gene was found to be associated with reduced risk of AR in this study, which was not associated with ESRD. G is wild type and A is variant. One previous study reported that the rs111568835 is associated with an increased incidence of rheumatoid arthritis in the Chinese population [30]. In an EGF gene promoter polymorphism study, rs11568835 was associated with a decreased risk of gastric cancer as haplotypes made with two other promoter SNPs of EGF gene (rs4444903 and rs3756261) in the Chinese population [31]. The EGF gene contains an atypical TATA box, polypurine-rich motifs, and consensus binding sequences for many transcription factors like AP-1, Sp-1, NF-kB, etc [32,33]. Genetic variants in the EGF promoter region may contribute to the differences of EGF expression and the subsequent disease susceptibility among individuals. Wang Y, *et al.* studied the association between EGF promoter SNPs and the risk of breast cancer. They did not find significant association between promoter SNPs of the EGF gene and the risk of breast cancer, but they found that plasma EGF level was significantly higher in the AA genotype of rs11568835 than that in the GG genotype [34]. The AA and AG genotype of rs11568835 were associated with decreased risk of AR in our study. We did not measure the amount of tissue or blood EGF, but it is possible that rs11568835 increased the amount of EGF expression and thus, showed a protective effect against AR.

Another SNP (rs1050171; Q787Q) found to be associated with increased risk of AR but not with ESRD is located in exon 20 region of the EGFR gene. In the previous studies, the presence of this mutation was associated with worse prognosis in colorectal cancer and lung squamous cell carcinoma than that in the wild type [35,36]. As a synonymous variant, rs1050171 does not alter the amino acid sequence and structure of EGFR. However, synonymous mutations can delay mRNA translation and reduce protein production [37]. In an *in vitro* study using squamous cell carcinoma of the head and neck (SCCHN) cell lines with rs1050171, AG genotype of rs1050171 showed significantly increased EGFR mRNA half-life and decreased EGFR protein levels when compared with the GG genotype [38]. Thus, one possible explanation about the association between the increased risk of AR and AG genotype of rs1050171 found in this study is that rs1050171 may increase the susceptibility to AR by delaying EGFR mRNA translation.

Among the 63 patients with AR, renal biopsy was done in 31 cases. A total of 16 cases of T-cell-mediated rejection and 2 cases of antibody-mediated rejection were confirmed by biopsies. The clinical diagnosis of AR was made as previously described. The rest of the biopsy specimens, which were not enough to be confirmed as AR were five cases of interstitial inflammation, three cases of tubular atrophy, three cases of acute tubular necrosis, one case of glomerular sclerosis, one case of cytoplasmic vacuolization, and one case with no specific abnormal lesion.

The low heterogeneity and low frequency of minor homozygotes in our study subjects could affect the results. Our study lacked matched controls involving other comorbidities that are supposed to be associated with EGF and EGF receptor polymorphisms, which might influence our findings. Another limitation of our study is the small size of the cohort and the retrospective nature of this study. Nevertheless, this is the first report to find that rs11568835 may be associated with lower susceptibility to AR, rs1050171 may be associated with higher susceptibility to AR, and rs11569017 may be associated with higher susceptibility to ESRD in the Korean population. Additional studies involving a larger number of cases and other populations will be necessary to validate and confirm the observed associations. The relationship between polymorphisms in the donor and AR could also be one of the possible research directions, because EGF and EGFR are expected to have a protective effect against renal damage.

In conclusion, this study suggests that 1) the homozygote of exonic polymorphism of EGF gene, TT genotype of rs11569017, may have an association with higher susceptibility to ESRD, 2) the homozygote of promoter polymorphism of EGF gene, AA genotype of rs1156835, may have a protective association with AR, and 3) the heterozygote of exonic polymorphism of EGFR gene, GA genotype of rs1050171, may have an association with higher susceptibility to AR in the Korean population.
